# Seasonal and sexual variations in plasma concentrations of testosterone, 17β-estradiol, and progesterone in the endangered Beale’s Eyed Turtle, *Sacalia bealei*, in captivity

**DOI:** 10.7717/peerj.21279

**Published:** 2026-06-05

**Authors:** Hui Li, Han Yang, Ying-Nan Lu, Hai-Tao Shi, Liu Lin

**Affiliations:** 1 Ministry of Education Key Laboratory for Ecology of Tropical Islands, Key Laboratory of Tropical Animal and Plant Ecology of Hainan Province, College of Life Sciences, Hainan Normal University, Haikou, China; 2 Qianwei Middle School, Xishan District, Kunming, China; 3 Tongliao Ecology and Environment Bureau, Inner Mongolia, China

**Keywords:** *Sacalia bealei*, Testosterone, 17β-estradiol, Progesterone, Seasonally, Reproduction, Captivity

## Abstract

Seasonally breeding species exhibit annual gonadal hormone fluctuations that synchronize with reproductive activity and ecology. However, the reproductive physiology of the Beale’s Eyed Turtle (*Sacalia bealei*), a critically endangered turtle species endemic to southeastern China, remains profoundly undocumented—a knowledge gap that is imperative for its recovery. Herein, we measured plasma concentrations of testosterone (T), 17β-estradiol (E2), and progesterone (P4) in captive *S. bealei* over a one-year period using enzyme-linked immunosorbent assay. Both male and female *S. bealei* display distinct seasonal reproductive cycles. Males showed a bimodal T profile (peaks in August and May), likely associated with spermatogenesis and mating in early autumn and mating in spring, suggesting a dissociated strategy with postnuptial spermatogenesis. A small rise in E2 and P4 levels in August may regulate male spermiogenesis. Females exhibited a single E2 peak in April, associated with Type I vitellogenesis and a single annual clutch, with T peaks in April and August, likely corresponding to ovarian growth and recrudescence, respectively, and P4 peaks in June linked to ovulation. Positive correlations between hormones suggest synergistic regulation. The data from this study provide a reference for future research regarding the reproductive cycle of this turtle and also allow reproductive management while in captivity.

## Introduction

Reproductive cycles in ectothermic animals are predominantly seasonal and often initiated by the changes in environmental factors such as temperature, photoperiod, and rainfall (Donini et al., 2021; Hosseinzadeh Sahafi et al., 2020). Like other ectotherms, turtles have reproductive cycles that follow similar cues (Allman et al., 2019). Turtles adapt their physiology and behavior in response to seasonal environmental shifts to maintain homeostasis, for instance, water and energy balance, among other processes (Hosseinzadeh Sahafi et al., 2020). These adaptations are largely regulated by steroid hormones that exhibit seasonal fluctuations, allowing turtles to anticipate and adjust to upcoming environmental changes like migration, hibernation, or reproduction (Al-Habsi et al., 2006; Carbajal et al., 2024).

In reptiles, gonadal sex steroids testosterone (T), 17β-estradiol (E2), and progesterone (P4) play key roles in growth, immune function, and reproduction, regulated by the hypothalamic-pituitary-gonadal (HPG) axis (Grant et al., 2016; Jalabert, 2005). Generally, T and E2 are associated with gonadal growth and gamete development, and P4 is linked to follicular ovulation (Branson, Atkinson & Ramos, 2016). Within turtles, there have been few species studied with sufficient depth to give us a general idea of the reproductive endocrinology. To date, turtles in which the endocrinology of reproduction has been characterized in wild populations of *Graptemys flavimaculata* (Shelby et al., 2000), *Gopherus flavomarginatus* (González Trápaga, Aguirre & Adest, 2000), *Graptemys gibbonsi* (Graham et al., 2015), *Gopherus polyphemus* (Allman et al., 2019), and *Macrochelys temminckii* (Rostal et al., 2023), as well as in captive specimens of *Lepidochelys kempi* (Rostal et al., 1998a), *Geochelone nigra* (Rostal et al., 1998b), *Caretta caretta* (Kakizoe et al., 2010), *Chelonoidis nigra* (Branson, Atkinson & Ramos, 2016), *Podocnemis expansa* (Freneau et al., 2017), and *M. temminckii* (Thompson et al., 2023). Additionally, the wild and captive population of *Testudo hermanni* has been examined (Huot-Daubremont et al., 2003). Analyses of these turtles’ hormonal cycles have revealed both consistent timing of reproductive events and their association with sex hormone levels. Sex steroids affect every physiological process, making their proper regulation essential for vertebrate health and reproductive success (Karimi et al., 2014). Detailed information on reproductive hormone patterns and interactions in turtles is vital to accurately assess their annual reproductive potential as well as to elucidate the mechanisms underlying gametogenesis, gonadal steroid production, and reproductive behaviors (Branson, Atkinson & Ramos, 2016; Kurita et al., 2025).

Reptilian testicular cycles generally display two primary patterns, with prenuptial spermatogenesis (associated) occurring when spermatogenesis immediately precedes mating and postnuptial spermatogenesis (dissociated) occurring when testicular recrudescence follows the breeding season, accompanied by sperm storage in the epididymis after spermiogenesis ceases in autumn (Kuchling, 1999; Van Wyk, 1995). Most temperate-zone freshwater and terrestrial turtles exhibit postnuptial spermatogenesis (Blanvillain, Owens & Kuchling, 2011; Graham et al., 2015; Rostal et al., 2023; Thompson et al., 2023), whereas prenuptial cycles are common in tropical and subtropical species (Licht, Wood & Wood, 1985; Singh, 1977; Wibbels et al., 1990). Turtles also differ in their vitellogenesis process (Mayor et al., 2023). Elevated E2 facilitates type I vitellogenesis in spring, leading to the onset and completion of vitellogenesis for that reproductive year (Aldridge, 1979; Taylor & Schuett, 2004). Subsequently, E2 levels decline while P4 surges around ovulation in spring or early summer (Currylow et al., 2013). Additionally, in late summer and early fall, E2 rises to drive type II vitellogenesis, supporting continued vitellogenesis and follicular enlargement for the next season’s initial clutch (Aldridge, 1979; Graham et al., 2015). Quantifying annual sex steroid hormone concentrations in turtles is a useful and practical way for clarifying their reproductive cycle types (Allman et al., 2019).

Beale’s Eyed Turtle (*Sacalia bealei*) is an endemic freshwater turtle in southeastern China, primarily distributed in Anhui, Fujian, Jiangxi, Hunan, Guizhou, Guangxi, Guangdong, and Hong Kong (Lin et al., 2022; Shi et al., 2008a). It is categorized as endangered on the IUCN Red List of Threatened Species and Appendix II of CITES due to multiple threats such as habitat degradation or loss, heavy poaching, and illegal trade (IUCN, 2000; Lin et al., 2018). *S. bealei* inhabits ponds, streams, lakes, paddy fields, and woodland habitats (Hu et al., 2016). Its breeding period in the wild occurs from April to June and in November, with egg-laying from late April to mid-June. The non-breeding period lasts from July to October, followed by an extended state of hibernation from December to March (Yuan et al., 2025a). Research on reproduction is necessary to protect endangered species. Previous studies of *S. bealei* mainly focus on its taxonomy and distribution (Lin et al., 2022; Shi et al., 2008b; Stuart & Parham, 2007), genetics (Li, Gong & Hua, 2014; Lin et al., 2020), sexual dimorphism (Lin et al., 2017), reproductive biology (Lin et al., 2018), species’ habitat use (Hu et al., 2016; Yuan et al., 2025a; Yuan et al., 2025b), and foraging ecology (Sung et al., 2021). However, the seasonal hormonal profiles remain completely unknown. Here, we quantified annual sex steroids testosterone (T), 17β-estradiol (E2), and progesterone (P4) of both sexes of captive *S. bealei* to ascertain reproductive cycles. The present study will give new insights into the relationship between reproductive physiology and behavior in this threatened turtle species and may aid in captive management and reproduction.

## Materials and Methods

### Study animal and blood plasma collection

A total of 12 healthy captive adult *S. bealei* (six males, plastron length 102.90–111.01 mm; six females, plastron length 119.08–134.07 mm) were obtained from the zoology laboratory of Hainan Normal University. The body mass was weighed, and carapace length, carapace width, plastron length, body height, and tail length were measured for all individuals following the methodology described by Lin et al. (2017) (Table S1). The turtles were housed in two indoor cement pools (200 cm × 80 cm × 80 cm) with a glass front, each containing three males and three females. Each pool had a water depth of 15–20 cm and contained an inverted basin with a diameter of 40 cm serving as shelter and a basking platform. Water changes were carried out once a week in spring, autumn, and winter, and every two days in summer. The process involved first draining the turbid water containing residual feed and feces from the bottom, followed by adding fresh water. Furthermore, the pools were disinfected every 2–3 weeks with 0.27–0.33% povidone-iodine solution. The turtles were fed at noon during spring, autumn, and winter and twice daily at 8:00 AM and 6:00 PM in summer, with a diet consisting of shrimp, chopped fish, apples, pears, and vegetables, at a daily ration equivalent to 0.27–0.33%. of their body mass. The photoperiod was the same as outside, and UVB lamps were used during the daytime. Air temperature was recorded daily. Turtles were maintained in captivity after the conclusion of the study.

Blood samples were collected monthly from all animals between March 2017 and February 2018. Prior to sampling, the animals were anesthetized by propofol (10 mg/mL) (Kristensen et al., 2023). Then, approximately 1 mL of blood was rapidly collected from the jugular vein into heparinized tubes and immediately centrifuged at 3,000 rpm for 5 min. The plasma was subsequently aliquoted into microfuge tubes and stored at −20 °C until further analysis. The animal care and experimental protocols have been reviewed and approved by the Animal Research Ethics Committee of Hainan Provincial Education Center for Ecology and Environment, Hainan Normal University (No. HNECEE-2014-003).

### Hormone analysis

Plasma testosterone (T), 17β-estradiol (E2), and progesterone (P4) were measured through an enzyme-linked immunosorbent assay (ELISA) using a commercially available kit (Beijing North Biotechnology Research Institute Co., Ltd.). The antibodies of T, E2, and P4 were the rabbit anti-testosterone antibody, rabbit anti-17β-estradiol antibody, and rabbit anti-progesterone antibody, respectively. The experiments were conducted following the manufacturer’s instructions. First, all the reagents and samples were brought to room temperature (21–26 °C) prior to use for 20 min. Next, standard, sample, and blank wells were prepared separately on the Microelisa Strip Plate in duplicate. Each standard well received 50 μL of standards at different concentrations. Each sample well received 40 μL of dilution buffer and 10 μL of the sample with gentle mixing. Blank wells were prepared in the same way as sample wells, except that both the sample and the HRP-conjugated reagent were omitted. Then, 100 μL of HRP-conjugated reagent was added to the standard and sample wells, the plate was sealed with the closure plate membrane, and it was incubated at 37 °C for 1 h. After incubation, the plate was washed five times with wash buffer by discarding the liquid, blotting on absorbent paper, filling wells with wash buffer for 1 min, and repeating the process. Subsequently, 50 μL each of substrate solutions A and B were added to each well, followed by incubation at 37 °C for 15 min in the dark. The reaction was terminated with 50 μL stop solution, and the optical density (OD) at 450 nm was immediately measured using a microplate reader. Finally, mean OD values were calculated for the standard, sample, and blank wells, with the standard and sample OD values corrected by subtracting the mean blank value. A standard curve was fitted in Excel using the corrected standard OD values *versus* concentration, from which sample concentrations were derived and subsequently multiplied by the dilution factor to yield the actual concentrations. Intra- and inter-assay coefficients of variation were <10% and <15%, respectively, for all assays.

### Statistical analyses

Normality and variance homogeneity were verified using the Kolmogorov–Smirnov and Levene’s tests, respectively. The variation in individual hormone levels in each sex by month was determined by one-way analysis of variance (ANOVA) followed by Tukey’s post hoc tests. To test for differences in hormone levels between two sexes within each month, we ran the analysis of covariance (ANCOVA) with hormone levels as the dependent variable, sex as the fixed term, and carapace length as a covariate to remove its effect (Graham et al., 2015). The relationship between the circulating values of the sex steroid hormones in males and females was explored using the Pearson correlation. Analyses were performed in SPSS 26.0 (SPSS Inc.), and figures were generated using OriginPro 2025 software. Data are presented as means ± SEM, with significance set at *p* < 0.05.

## Results

**Figure 1:**
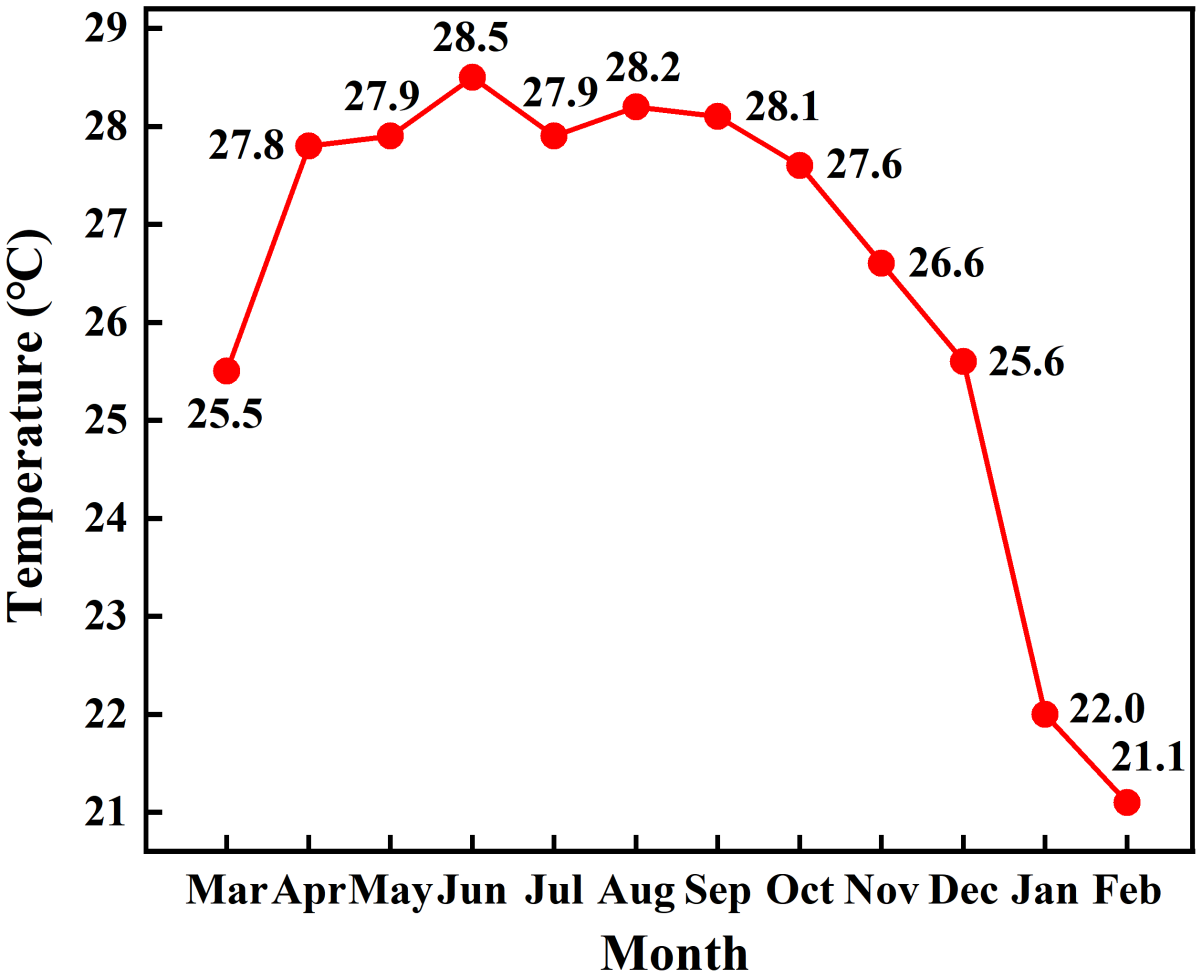
The change of annual temperature in the captivity room.

During the study period, the mean annual temperature in the captivity room was 26.4 °C. The lowest mean temperature (21.1 °C) was recorded in February 2018, while the highest (28.5 °C) occurred in June 2017 (Fig. 1).

### Annual changes of plasma T, E2, and P4 in males and females

**Figure 2:**
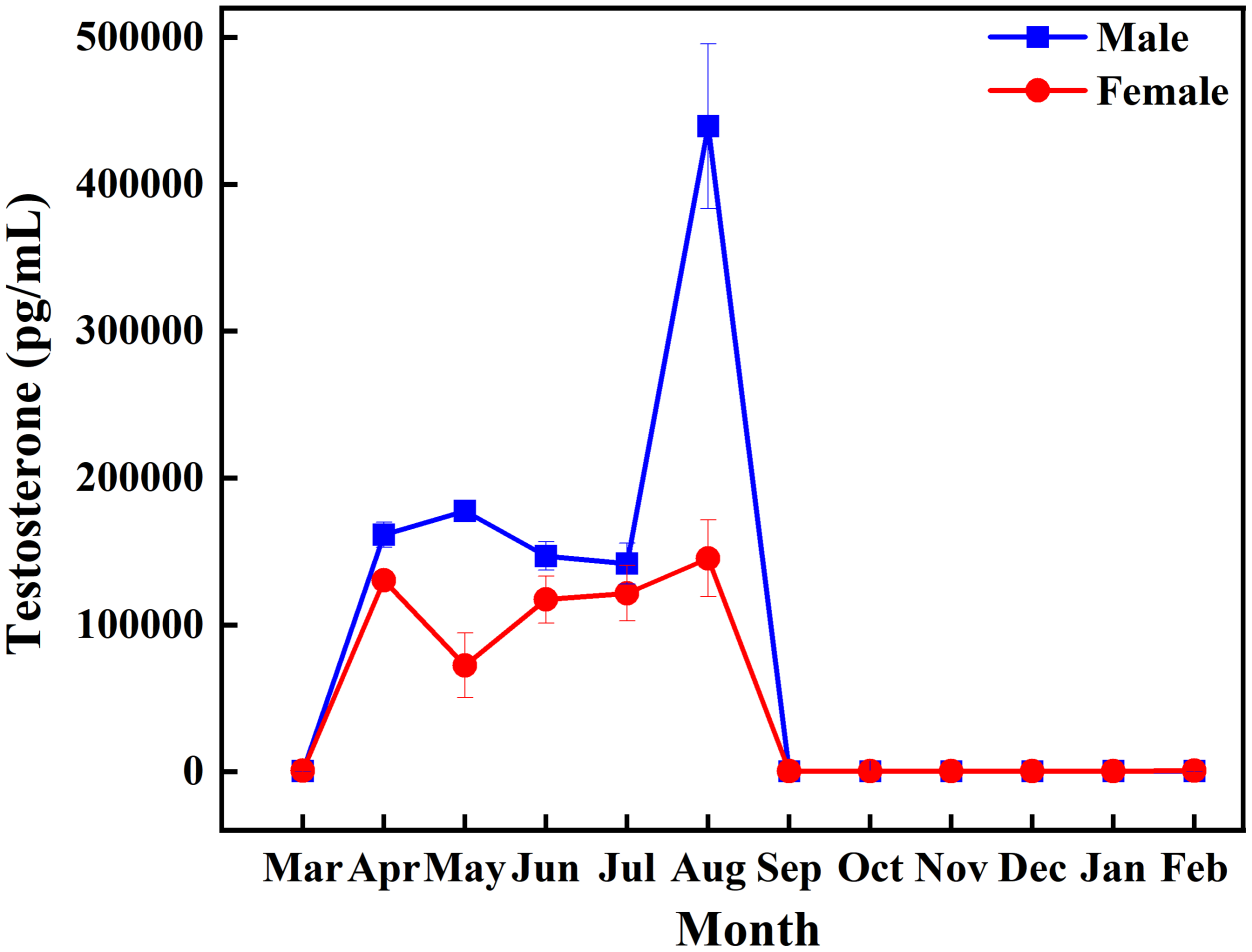
Changes in mean monthly plasma testosterone concentrations in males (square symbol, blue) and females (circular symbol, red) of *Sacalia bealei* throughout the annual reproductive cycle. Dots and vertical bars represent the means ± SEM.

T concentrations in male and female *S*. *bealei* began to rise from March to April. Subsequently, male concentrations increased gradually in May and decreased progressively from June to July, whereas females showed the reverse pattern to males during this period. Both sexes then increased sharply and peaked in August, followed by a gradual decrease starting in September, with peak values reaching 439,607.43 ± 56,138.01 pg/mL in males and 145,295.78 ± 26,117.92 pg/mL in females. In other months, both sexes maintained low and stable serum T concentrations (Fig. 2).

**Figure 3:**
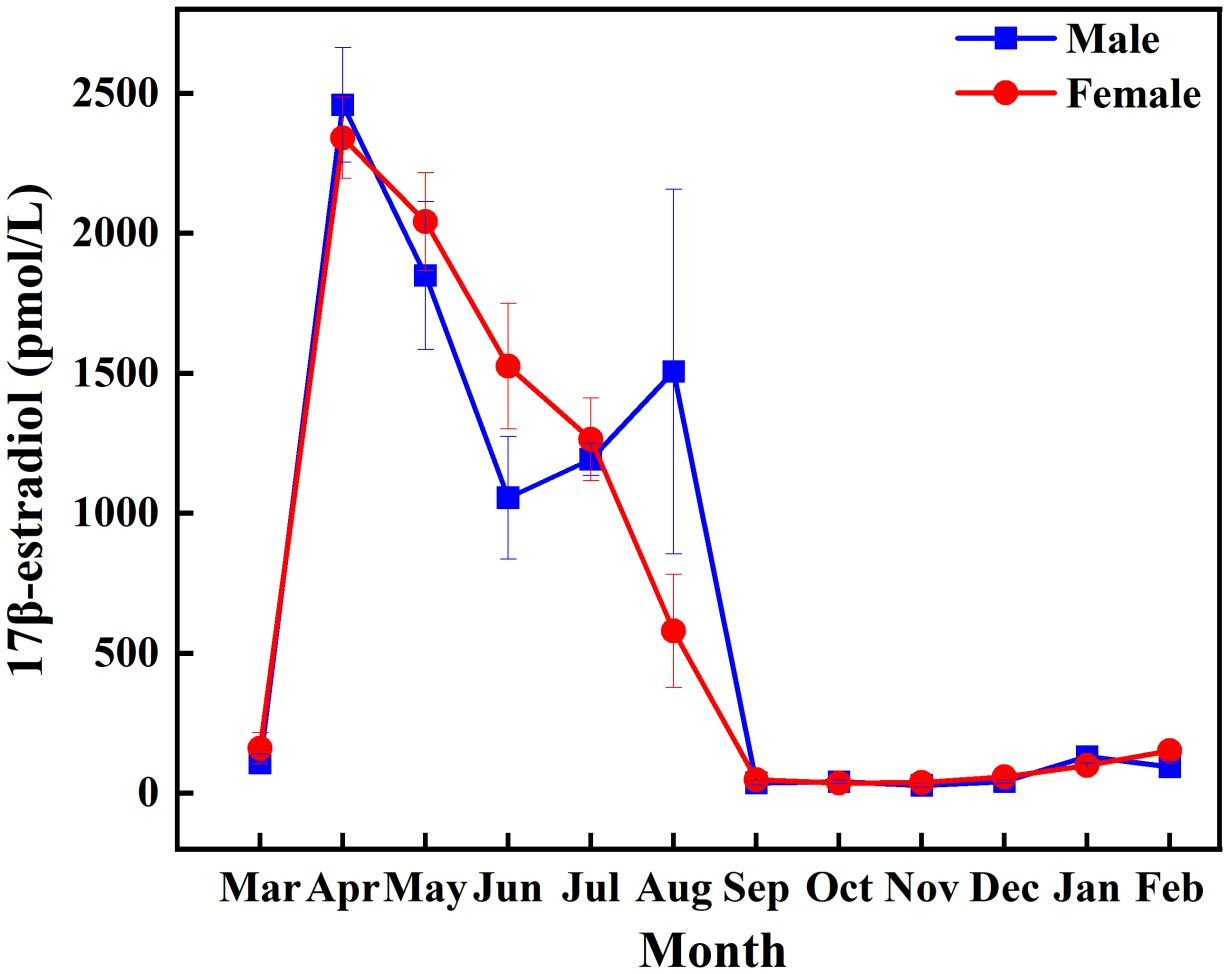
Changes in mean monthly plasma 17β-estradiol concentrations in males (square symbol, blue) and females (circular symbol, red) of *Sacalia bealei* throughout the annual reproductive cycle. Dots and vertical bars represent the means ± SEM.

E2 concentrations in male and female *S. bealei* increased sharply from March to April, peaking in April at 2,458.66 ± 204.54 pmol/L and 2,341.05 ± 145.45 pmol/L, respectively. Next, male concentrations decreased from May to June and then increased again from July to August, with a secondary peak in August at 1,505.91 ± 651.84 pmol/L before returning to minimum concentrations in September. Male concentrations remained low from October to December, with a slight increase in January followed by a slight fall in February. While female concentrations declined gradually to minimal values from May through September, they remained low from October to December and increased progressively from January to February (Fig. 3).

**Figure 4:**
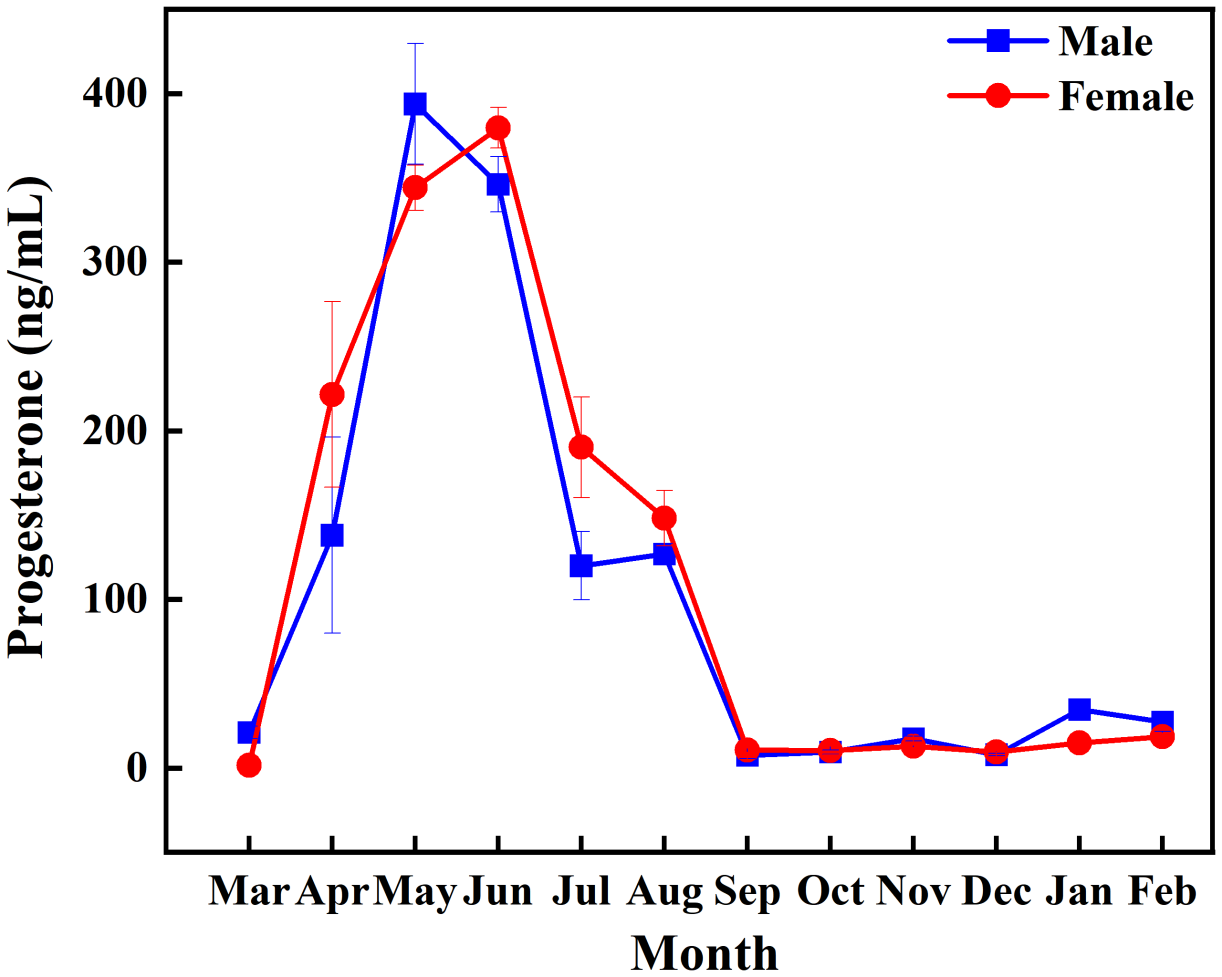
Changes in mean monthly plasma progesterone concentrations in males (square symbol, blue) and females (circular symbol, red) of *Sacalia bealei* throughout the annual reproductive cycle. Dots and vertical bars represent the means ± SEM.

P4 concentrations in male *S. bealei* increased sharply from March to May, peaking in May at 393.80 ± 35.77 ng/mL, declined progressively from June to July, and rose again in August before decreasing back to minimal concentrations in September, and then maintained a low level from October to December with a slight increase in January followed by a slight drop in February. However, concentrations in females increased sharply from March to June, peaking in June at 379.71 ± 12.08 ng/mL, declined progressively to minimal levels from July to September, maintained a low level from October to December, and then rose slowly from January to February (Fig. 4).

### Differences in plasma T, E2, and P4 levels between months and sexes

T, E2, and P4 varied seasonally in males (T: *df* = 11, *F* = 61.005, *P* < 0.001; E2: *df* = 11, *F* = 15.234, *P* < 0.001; P4: *df* = 11, *F* = 39.402, *P* < 0.001). The peak T level in males in August was significantly higher than in all other months (*P* < 0.05; Table 1; Table S2), whereas E2 and P4 levels were significantly higher from April to August compared with other months (*P* < 0.05; Tables 2–3; Tables S2–S4). Significant seasonal variation was also observed in females (T: *df* = 11, *F* = 26.089, *P* < 0.001; E2: *df* = 11, *F* = 52.820, *P* < 0.001; P4: *df* = 11, *F* = 51.615, *P* < 0.001). T concentrations in females were significantly higher in April and from June to August than in the remaining months (*P* < 0.05; Table 1; Table S5), while a similar pattern to males was seen for E2 and P4, with higher levels from April to August (*P* < 0.05; Tables 2–3; Tables S6–S7).

**Table 1:** Differences in plasma testosterone levels between months and sexes.

**Month**	**Testosterone (pg/mL)**		***df***	***F***	**Intersex** ***p*** **-value**
	**Males (Mean ± SEM)**	**Females (Mean ± SEM)**			
2017-Mar	362.50 ± 38.42^c^	583.77 ± 36.98^c^	1	6.828	0.028^*^
2017-Apr	161,352.35 ± 8,464.99^b^	130,346.74 ± 4,203.83^a^	1	1.873	0.204
2017-May	177,495.86 ± 3,826.62^b^	72,336.59 ± 21,968.93^b^	1	7.512	0.023^*^
2017-Jun	146,858.99 ± 9,568.50^b^	117,137.87 ± 15,911.81^a^	1	0.240	0.636
2017-Jul	141,734.66 ± 13,993.71^b^	121,530.16 ± 18,891.81^a^	1	1.196	0.302
2017-Aug	439,607.43 ± 56,138.01^a^	145,295.78 ± 26,117.92^a^	1	5.843	0.039^*^
2017-Sep	307.16 ± 17.54^c^	318.08 ± 12.43^c^	1	0.000	0.994
2017-Oct	327.70 ± 42.50^c^	304.11 ± 11.31^c^	1	0.528	0.486
2017-Nov	268.90 ± 30.99^c^	358.45 ± 18.74^c^	1	1.891	0.202
2017-Dec	283.80 ± 7.54^c^	242.49 ± 22.88^c^	1	2.213	0.171
2018-Jan	444.08 ± 47.56^c^	481.14 ± 30.13^c^	1	0.845	0.382
2018-Feb	455.26 ± 27.17^c^	539.47 ± 12.13^c^	1	4.311	0.068

**Notes.**

Different lowercase letters indicate significant differences between months within the same sex (Tukey’s test, *P* <0.05).

The symbol * indicates a significant difference between sexes for a given month (ANCOVA, *P* < 0.05), and significant values are shown in bold.

**Table 2:** Differences in plasma 17β-estradiol levels between months and sexes.

**Month**	**17β-estradiol (pmol/L)**		***df***	***F***	**Intersex** ***p*** **-value**
	**Males (Mean ± SEM)**	**Females (Mean ± SEM)**		
2017-Mar	107.51 ± 32.07^d^	159.72 ± 56.12^d^	1	0.030	0.865
2017-Apr	2,458.66 ± 204.54^a^	2,341.05 ± 145.45^a^	1	0.065	0.805
2017-May	1,848.88 ± 263.51^ab^	2,041.03 ± 175.12^a^	1	1.036	0.335
2017-Jun	1,055.62 ± 218.70^c^	1,524.99 ± 224.30^b^	1	8.593	0.017^*^
2017-Jul	1,193.18 ± 57.47^bc^	1,263.89 ± 148.51^b^	1	0.074	0.791
2017-Aug	1,505.91 ± 651.84^bc^	580.15 ± 202.29^c^	1	0.720	0.418
2017-Sep	36.77 ± 6.44^d^	48.03 ± 5.94^d^	1	0.719	0.418
2017-Oct	41.05 ± 6.02^d^	36.47 ± 4.52^d^	1	0.042	0.841
2017-Nov	28.36 ± 6.59^d^	37.37 ± 3.57^d^	1	0.051	0.826
2017-Dec	40.98 ± 5.08^d^	58.99 ± 3.81^d^	1	1.200	0.302
2018-Jan	131.41 ± 24.10^d^	98.98 ± 16.88^d^	1	1.479	0.255
2018-Feb	93.90 ± 19.68^d^	152.49 ± 12.63^d^	1	5.983	0.037^*^

**Notes.**

Different lowercase letters indicate significant differences between months within the same sex (Tukey’s test, *P* < 0.05).

Please revise this sentence to: The symbol * indicates a significant difference between sexes for a given month (ANCOVA, *P* < 0.05), and significant values are shown in bold.

**Table 3:** Differences in plasma progesterone levels between months and sexes.

**Month**	**Progesterone (ng/mL)**		***df***	***F***	**Intersex** ***p*** **-value**
	**Males (Mean ± SEM)**	**Females (Mean ± SEM)**			
2017-Mar	21.05 ± 3.65^c^	1.64 ± 0.65^d^	1	7.450	0.023^*^
2017-Apr	138.07 ± 58.08^b^	221.56 ± 55.13^b^	1	0.111	0.747
2017-May	393.80 ± 35.77^a^	344.18 ± 13.40^a^	1	0.000	0.991
2017-Jun	346.22 ± 16.38^a^	379.71 ± 12.08^a^	1	0.114	0.744
2017-Jul	119.98 ± 20.31^b^	190.23 ± 29.92^bc^	1	6.956	0.027^*^
2017-Aug	126.87 ± 6.39^b^	148.05 ± 16.38^c^	1	3.526	0.093
2017-Sep	7.43 ± 1.80^c^	10.60 ± 1.27^d^	1	0.003	0.956
2017-Oct	9.43 ± 1.30^c^	10.31 ± 2.46^d^	1	0.641	0.444
2017-Nov	17.37 ± 1.40^c^	12.83 ± 0.72^d^	1	3.547	0.092
2017-Dec	7.91 ± 0.60^c^	9.62 ± 0.32^d^	1	1.780	0.215
2018-Jan	34.57 ± 5.83^c^	14.89 ± 2.74^d^	1	2.824	0.127
2018-Feb	27.18 ± 5.53^c^	18.61 ± 5.09^d^	1	0.043	0.840

**Notes.**

Different lowercase letters indicate significant differences between months within the same sex (Tukey’s test, *P* < 0.05).

The symbol * indicates a significant difference between sexes for a given month (ANCOVA, *P* < 0.05), and significant values are shown in bold.

Differences in plasma concentrations of three hormones (T, E2, and P4) were detected between males and females, with T significantly greater in males than in females in May and August (May: *df* = 1, *F* = 7.512, *P* < 0.05; August: *df* = 1, *F* = 5.843, *P* < 0.05) but greater in females in March (*df* = 1, *F* = 6.828, *P* < 0.05; Table 1); with E2 levels significantly greater in females than in males in both June and February (June: *df* = 1, *F* = 8.593, *P* < 0.05; February: *df* = 1, *F* = 5.983, *P* < 0.05; Table 2); and with P4 concentrations significantly higher in males than in females in March (*df* = 1, *F* = 7.450, *P* < 0.05) but higher in females in July (*df* = 1, *F* = 6.956, *P* < 0.05; Table 3).

### Correlations of plasma T, E2, and P4 in males and females

In males, plasma concentrations of T–E2, T–P4, and E2–P4 showed significant positive correlations (T–E2: *r* = 0.690; T–P4: *r* = 0.480; E2–P4: *r* = 0.584; all *P* < 0.001). Similarly, in females, plasma concentrations of T–E2, T–P4, and E2–P4 were also positively correlated (T–E2: *r* = 0.680; T–P4: *r* = 0.712; E2–P4: *r* = 0.780; all *P* < 0.001).

## Discussion

It has been confirmed that stress from prolonged captivity suppresses sex steroid secretion and affects reproductive activities in turtles, so studies of hormonal cycles based on captive animals should be evaluated with caution (Huot-Daubremont et al., 2003; Mahmoud & Licht, 1997).

Male *S. bealei* showed a dual peak in T, a pattern similar to other bimodally breeding turtles (Currylow et al., 2013; Freneau et al., 2017). High T concentration in male turtles coincides with spermatogenesis during fall, as seen in *Sternotherus odoratus* (McPherson et al., 1982). In addition, multiple studies have documented turtles mating behaviors in fall (Graham et al., 2015; Naimi et al., 2024), with spermatozoa having been successfully recovered from oviducts of various species during the autumn (Xiangkun et al., 2008; Zhang et al., 2015). Mating in wild *S. bealei* is reported to occur in April and November (Yuan et al., 2025a). Accordingly, the maximal T levels were observed in early autumn (August) following the breeding season, likely linked to gonadal recrudescence (postnuptial spermatogenesis) and mating. Spermatozoa produced in the fall are stored in the epididymal canals over winter, becoming available for fertilization in the subsequent spring mating season (Allman et al., 2019), while autumn mating enables oviductal sperm storage in females until spring ovulation and fertilization (Gist et al., 2001). Our study also identified elevated T levels during the spring mating, with parallel changes in male T and female E2 levels in April, indicating synchronized reproductive readiness. This suggests a potential dissociation between mating activity and androgen biosynthesis in *S. bealei*, contrasting with some turtle species where T peaks directly correspond with mating (Allman et al., 2019; Graham et al., 2015; Lagarde et al., 2003). Male sexual activity is modulated by intricate interactions between T levels and environmental factors (Sereau et al., 2010). In common musk turtles (*S. odoratus*), experimental evidence demonstrates that elevated T facilitates sexual behavior initiation and maintenance, though its effects exhibit seasonal variation and are primarily photoperiod-dependent, with temperature playing a secondary regulatory role (Mendonça, 1987). Therefore, elevated temperature in the spring (March to May) likely serves as the trigger for initiating male mating behavior in this species.

The profile of plasma E2 in male *S. bealei* was similar to that of plasma T during both active periods. In male red-eared sliders (*Trachemys scripta elegans*), circulating E2 levels increase specifically during spermatogenesis, where E2 has been shown to induce spermatogonia proliferation in the testis (Li et al., 2023). The peak E2 level in August likely regulates spermiogenesis by directly binding to estrogen receptors on spermatids and spermatozoa, thereby maintaining their viability and preventing spermatid death (Pewhom, Supapakorn & Srakaew, 2022). Further research is needed to clarify E2’s specific regulatory mechanisms in *S*. *bealei* spermatogenesis. Several studies have reported that E2 in a synergistic way with T may influence territoriality, courtship, copulation, and testicular growth in male reptiles (Al-Amri et al., 2013; Maistrelli et al., 2022).

P4 may serve as a substrate for T and E2 synthesis in male *S*. *bealei*, which could account for the positive correlations between circulating P4 and T/E2 levels (Norris, 2007). Additionally, evidence indicates that P4 can directly bind to germ cell progesterone receptors across developmental stages, potentially regulating sexual maturation and synergizing with T to modulate reproductive behaviors (Crews & Moore, 2005). Like serum T profiles, circulating P4 levels increased incrementally in August, suggesting its involvement in the regulation of spermiogenesis and spermiation, as evidenced by the enhanced immunoreactivity of progesterone receptors in the testes with advancing spermatogenic stages of male green turtles (Otsuka et al., 2008).

In female *S. bealei*, the seasonal change in T is similar to that reported in other female turtles (McPherson et al., 1982). Two maxima occur, one at the beginning of the annual activity cycle, which can be related to ovarian growth prior to oviposition (Thompson et al., 2023), and the second associated with ovarian recrudescence and vitellogenesis of the following year’s clutch (Rostal et al., 2023; Wolfe, Donini & Valverde, 2023). As T is a known precursor for E2 synthesis, the increase in T observed in April, which paralleled a concomitant elevation in E2, may primarily support E2 synthesis (Blanvillain, Owens & Kuchling, 2011). Alternatively, some propose that increasing T levels may initiate breeding behaviors and potentially stimulate male-attracting pheromone production (Al-Habsi et al., 2006; Rubin et al., 2023). Elevated T levels have been linked to female receptivity and mating behavior across multiple turtle species, including *Gopherus agassizii* (Rostal et al., 1994), *L. kempi* (Rostal et al., 1998a), and *G. polyphemus* (Allman et al., 2019).

A peak in female E2 in April may be implicated in several early reproductive activities, such as sexual receptivity and pheromone production (Parker & Mason, 2012), vitellogenesis and oviduct development (Herbst et al., 2003; Sifuentes-Romero et al., 2006). Furthermore, in those turtles with multiple clutches, E2 peaks numerous times during the summer nesting season (Currylow et al., 2013). By contrast, the single E2 peak in our data indicates that *S*. *bealei* likely lays a single clutch of eggs in one season. This strategy may enhance offspring survival because hatchlings emerge under favorable conditions with warmer temperatures, abundant food, and sufficient time to grow and accumulate resources for the forthcoming winter (Lovich et al., 2014). Other freshwater turtle species are also documented to produce single clutches (*e.g.*, *G. flavimaculata and Glyptemys muhlenbergii*) (Lovich et al., 2014; Shelby et al., 2000). E2 begins to decline in May, indicating oocytes undergoing maturation and hydration signal vitellogenesis completion and spawning season onset (Ghosh et al., 2016; Zupa et al., 2017). However, we lacked samples showing post-vitellogenesis or recent-spawning hormone profiles in *S*. *bealei*, preventing assessment of post-spawning E2 patterns. Future studies measuring pre- and post-spawning *S. bealei* hormone levels would clarify E2 dynamics in this species.

P4, secreted by the corpora lutea, is involved in follicular growth, ovulation, egg production, and the reduction of oviductal movement during egg shelling (Blanvillain, Owens & Kuchling, 2011). Elevated P4 levels have been reported during mating and nesting periods in captive female *Chelonia mydas* (Licht et al., 1979) and during ovulation and egg shelling in *M*. *temminckii* (Thompson et al., 2023). The peak in plasma P4 observed in June in female *S*. *bealei* coincides with the peak spawning period in the wild, suggesting an association with ovulation and nesting activity (Currylow et al., 2017; Yuan et al., 2025a). Progesterone also plays a role in the inhibition of vitellogenesis (Custodia-Lora, Novillo & Callard, 2004). The concurrence of elevated P4 and the highest yearly E2 levels suggests that follicular hypertrophy and atresia may occur simultaneously in *S*. *bealei*. Moreover, egg production in the endangered western swamp turtle (*Pseudemydura umbrina*) seems to be critically dependent on both temperature and food supply (Kuchling & Dejose, 1989). Thus, the maximal temperatures in the rearing room and twice-daily feeding in June may be correlated with elevated plasma P4 concentrations in female *S. bealei*.

### Conclusions

In this study, method validation procedures for ELISA, including dilutional linearity, parallelism, and matrix interference, were not performed in this non-model organism (Allen et al., 2015). Future endocrinology research on *S. bealei* should conduct these validations to ensure assay reliability. Several researchers have noted that a full understanding of reproductive endocrinology in turtles has been constrained by limited blood sampling, especially in the endangered and threatened species where sampling opportunities are inherently restricted (Blanvillain, Owens & Kuchling, 2011; Kuchling, 1999). Although this investigation is based on a small sample size, it gives us a greater understanding of the relationship between reproductive endocrinology and behavior in *S*. *bealei* and provides the first seasonal hormonal profiles for use in captive management. For instance, our findings demonstrate that *S*. *bealei* is a dissociated breeder, and thus separating males at the onset of the nesting season and delaying reintroduction until the subsequent mating season may enhance mating activity (Branson, Atkinson & Ramos, 2016). Additionally, female E2 production mainly in the spring indicates ongoing vitellogenesis, highlighting the necessity of sustained nutritional support and dietary supplementation for optimal follicular maturation (Cigler et al., 2024). P4 levels typically increase following ovulation due to corpus luteum activity, reaching maximal values during the interval preceding oviposition (Shelby et al., 2000). P4 in females peaked in June, which allowed us to acquire information from ovulation to oviposition for production and management. Future studies should incorporate ultrasound imaging to evaluate follicular growth, ovulation, testicular growth, and semen quality to enable comprehensive fertility assessments. In addition, histological examinations of the testes and ovaries would both disclose the actual gonadal cycle of *S. bealei* and corroborate the hormone profiles observed in this study (Rostal et al., 2023). Non-lethal biopsies of the gonads may provide the necessary evidence to confirm postnuptial spermatogenesis in males and Type I vitellogenesis in females. Captivity imposes stress that may have a profound effect on hormonal levels, so data from wild populations are needed to understand the basic reproductive biology of this species.

## Supplemental Information

10.7717/peerj.21279/supp-1 Supplemental Information 1 Supplemental tables

10.7717/peerj.21279/supp-2 Supplemental Information 2 Male *Sacalia bealei* Plasma Raw Data

10.7717/peerj.21279/supp-3 Supplemental Information 3 Female *Sacalia bealei* Plasma Raw Data

## Additional Information and Declarations

### Data Availability

The following information was supplied regarding data availability: The raw data are available in the Supplemental Files.
