# Supplementary material for: Seasonal and sexual variations in plasma concentrations of testosterone, 17β-estradiol, and progesterone in the endangered Beale’s Eyed Turtle, Sacalia bealei, in captivity
Source: PeerJ. 2026 Jun 5;14:e21279. doi: 10.7717/peerj.21279 (PMC13245426; doi:10.7717/peerj.21279)
Supplement: Supplemental Information 1 [file peerj-14-21279-s001.docx]

**Table S1 Morphometry of *Sacalia bealei.***

| **Specimen number** | **Sex** | **Body mass (g)** | **Carapace length (mm)** | **Carapace width (mm)** | **Plastron length (mm)** | **Body height (mm)** | **Tail length (mm)** |
| --- | --- | --- | --- | --- | --- | --- | --- |
| C1 | ♀ | 325 | 130.60 | 94.10 | 119.08 | 58.73 | 34.06 |
| C2 | ♀ | 332 | 137.06 | 105.05 | 130.70 | 53.03 | 38.09 |
| C4 | ♀ | 427 | 146.33 | 100.60 | 132.30 | 61.87 | 34.87 |
| C5 | ♀ | 416 | 152.08 | 102.30 | 126.08 | 52.73 | 35.03 |
| C6 | ♀ | 400 | 144.05 | 96.09 | 124.08 | 96.06 | 43.02 |
| C7 | ♀ | 547 | 160.30 | 103.50 | 134.07 | 65.70 | 41.06 |
| X4 | ♂ | 259 | 130.91 | 87.07 | 107.53 | 51.72 | 35.32 |
| X5 | ♂ | 265 | 127.01 | 84.10 | 108.07 | 43.04 | 33.04 |
| X6 | ♂ | 246 | 116.60 | 87.90 | 102.90 | 48.31 | 26.30 |
| X7 | ♂ | 308 | 131.72 | 92.06 | 110.10 | 52.04 | 41.04 |
| X8 | ♂ | 261 | 123.72 | 94.20 | 104.03 | 51.03 | 38.52 |
| X9 | ♂ | 258 | 127.72 | 87.43 | 111.01 | 56.00 | 41.04 |

**Table S2 Pairwise comparisons of plasma testosterone levels among months in males.**

| **Month** | **2017-Mar** | **2017-Apr** | **2017-May** | **2017-Jun** | **2017-Jul** | **2017-Aug** | **2017-Sep** | **2017-Oct** | **2017-Nov** | **2017-Dec** | **2018-Jan** | **2018-Feb** |
| --- | --- | --- | --- | --- | --- | --- | --- | --- | --- | --- | --- | --- |
| **2017-Mar** |  |  |  |  |  |  |  |  |  |  |  |  |
| **2017-Apr** | **< 0.001***** |  |  |  |  |  |  |  |  |  |  |  |
| **2017-May** | **< 0.001***** | 0.508 |  |  |  |  |  |  |  |  |  |  |
| **2017-Jun** | **< 0.001***** | 0.552 | 0.211 |  |  |  |  |  |  |  |  |  |
| **2017-Jul** | **< 0.001***** | 0.422 | 0.145 | 0.833 |  |  |  |  |  |  |  |  |
| **2017-Aug** | **< 0.001***** | **< 0.001***** | **< 0.001***** | **< 0.001***** | **< 0.001***** |  |  |  |  |  |  |  |
| **2017-Sep** | 0.998 | **< 0.001***** | **< 0.001***** | **< 0.001***** | **< 0.001***** | **< 0.001***** |  |  |  |  |  |  |
| **2017-Oct** | 0.999 | **< 0.001***** | **< 0.001***** | **< 0.001***** | **< 0.001***** | **< 0.001***** | 0.999 |  |  |  |  |  |
| **2017-Nov** | 0.997 | **< 0.001***** | **< 0.001***** | **< 0.001***** | **< 0.001***** | **< 0.001***** | 0.999 | 0.998 |  |  |  |  |
| **2017-Dec** | 0.997 | **< 0.001***** | **< 0.001***** | **< 0.001***** | **< 0.001***** | **< 0.001***** | 0.999 | 0.999 | 1.000 |  |  |  |
| **2018-Jan** | 0.997 | **< 0.001***** | **< 0.001***** | **< 0.001***** | **< 0.001***** | **< 0.001***** | 0.996 | 0.996 | 0.994 | 0.995 |  |  |
| **2018-Feb** | 0.997 | **< 0.001***** | **< 0.001***** | **< 0.001***** | **< 0.001***** | **< 0.001***** | 0.995 | 0.996 | 0.994 | 0.994 | 1.000 |  |

**Notes:**

Table values represent *p*-values from pairwise comparisons of plasma testosterone levels among months in males using one-way analysis of variance (ANOVA) followed by Tukey’s post hoc tests. *** *P* < 0.001, ** *P* < 0.01, * *P* *<* 0.05.

**Table S3 Pairwise comparisons of plasma 17β-estradiol levels among months in males.**

| **Month** | **2017-Mar** | **2017-Apr** | **2017-May** | **2017-Jun** | **2017-Jul** | **2017-Aug** | **2017-Sep** | **2017-Oct** | **2017-Nov** | **2017-Dec** | **2018-Jan** | **2018-Feb** |
| --- | --- | --- | --- | --- | --- | --- | --- | --- | --- | --- | --- | --- |
| **2017-Mar** |  |  |  |  |  |  |  |  |  |  |  |  |
| **2017-Apr** | **< 0.001***** |  |  |  |  |  |  |  |  |  |  |  |
| **2017-May** | **< 0.001***** | 0.056 |  |  |  |  |  |  |  |  |  |  |
| **2017-Jun** | **0.004**** | **< 0.001***** | **0.014*** |  |  |  |  |  |  |  |  |  |
| **2017-Jul** | **0.001**** | **< 0.001***** | **0.041*** | 0.662 |  |  |  |  |  |  |  |  |
| **2017-Aug** | **< 0.001***** | **0.004**** | 0.278 | 0.156 | 0.322 |  |  |  |  |  |  |  |
| **2017-Sep** | 0.822 | **< 0.001***** | **< 0.001***** | **0.002**** | **< 0.001***** | **< 0.001***** |  |  |  |  |  |  |
| **2017-Oct** | 0.833 | **< 0.001***** | **< 0.001***** | **0.002**** | **0.001**** | **< 0.001***** | 0.989 |  |  |  |  |  |
| **2017-Nov** | 0.802 | **< 0.001***** | **< 0.001***** | **0.002**** | **< 0.001***** | **< 0.001***** | 0.979 | 0.968 |  |  |  |  |
| **2017-Dec** | 0.833 | **< 0.001***** | **< 0.001***** | **0.002**** | **0.001**** | **< 0.001***** | 0.989 | 1.000 | 0.968 |  |  |  |
| **2018-Jan** | 0.939 | **< 0.001***** | **< 0.001***** | **0.005**** | **0.001**** | **< 0.001***** | 0.764 | 0.774 | 0.743 | 0.774 |  |  |
| **2018-Feb** | 0.965 | **< 0.001***** | **< 0.001***** | **0.003**** | **0.001**** | **< 0.001***** | 0.856 | 0.867 | 0.835 | 0.866 | 0.905 |  |

**Notes:**

Table values represent *p*-values from pairwise comparisons of plasma 17β-estradiol levels among months in males using one-way analysis of variance (ANOVA) followed by Tukey’s post hoc tests. *** *P* < 0.001, ** *P* < 0.01, * *P* *<* 0.05.

**Table S4 Pairwise comparisons of plasma progesterone levels among months in males.**

| **Month** | **2017-Mar** | **2017-Apr** | **2017-May** | **2017-Jun** | **2017-Jul** | **2017-Aug** | **2017-Sep** | **2017-Oct** | **2017-Nov** | **2017-Dec** | **2018-Jan** | **2018-Feb** |
| --- | --- | --- | --- | --- | --- | --- | --- | --- | --- | --- | --- | --- |
| **2017-Mar** |  |  |  |  |  |  |  |  |  |  |  |  |
| **2017-Apr** | **< 0.001***** |  |  |  |  |  |  |  |  |  |  |  |
| **2017-May** | **< 0.001***** | **< 0.001***** |  |  |  |  |  |  |  |  |  |  |
| **2017-Jun** | **< 0.001***** | **< 0.001***** | 0.120 |  |  |  |  |  |  |  |  |  |
| **2017-Jul** | **0.002**** | 0.551 | **< 0.001***** | **< 0.001***** |  |  |  |  |  |  |  |  |
| **2017-Aug** | **0.001**** | 0.712 | **< 0.001***** | **< 0.001***** | 0.820 |  |  |  |  |  |  |  |
| **2017-Sep** | 0.653 | **< 0.001***** | **< 0.001***** | **< 0.001***** | **< 0.001***** | **< 0.001***** |  |  |  |  |  |  |
| **2017-Oct** | 0.701 | **< 0.001***** | **< 0.001***** | **< 0.001***** | **0.001**** | **< 0.001***** | 0.948 |  |  |  |  |  |
| **2017-Nov** | 0.903 | **< 0.001***** | **< 0.001***** | **< 0.001***** | **0.001**** | **0.001**** | 0.743 | 0.793 |  |  |  |  |
| **2017-Dec** | 0.665 | **< 0.001***** | **< 0.001***** | **< 0.001***** | **< 0.001***** | **< 0.001***** | 0.987 | 0.960 | 0.755 |  |  |  |
| **2018-Jan** | 0.656 | **0.001**** | **< 0.001***** | **< 0.001***** | **0.006**** | **0.003**** | 0.372 | 0.408 | 0.571 | 0.380 |  |  |
| **2018-Feb** | 0.840 | **0.001**** | **< 0.001***** | **< 0.001***** | **0.003**** | **0.002**** | 0.515 | 0.558 | 0.746 | 0.525 | 0.807 |  |

**Notes:**

Table values represent *p*-values from pairwise comparisons of plasma progesterone levels among months in males using one-way analysis of variance (ANOVA) followed by Tukey’s post hoc tests. *** *P* < 0.001, ** *P* < 0.01, * *P* *<* 0.05.

**Table S5 Pairwise comparisons of plasma testosterone levels among months in females.**

| **Month** | **2017-Mar** | **2017-Apr** | **2017-May** | **2017-Jun** | **2017-Jul** | **2017-Aug** | **2017-Sep** | **2017-Oct** | **2017-Nov** | **2017-Dec** | **2018-Jan** | **2018-Feb** |
| --- | --- | --- | --- | --- | --- | --- | --- | --- | --- | --- | --- | --- |
| **2017-Mar** |  |  |  |  |  |  |  |  |  |  |  |  |
| **2017-Apr** | **< 0.001***** |  |  |  |  |  |  |  |  |  |  |  |
| **2017-May** | **< 0.001***** | **0.001**** |  |  |  |  |  |  |  |  |  |  |
| **2017-Jun** | **< 0.001***** | 0.448 | **0.012*** |  |  |  |  |  |  |  |  |  |
| **2017-Jul** | **< 0.001***** | 0.612 | **0.006**** | 0.800 |  |  |  |  |  |  |  |  |
| **2017-Aug** | **< 0.001***** | 0.391 | **< 0.001***** | 0.109 | 0.174 |  |  |  |  |  |  |  |
| **2017-Sep** | 0.988 | **< 0.001***** | **< 0.001***** | **< 0.001***** | **< 0.001***** | **< 0.001***** |  |  |  |  |  |  |
| **2017-Oct** | 0.987 | **< 0.001***** | **< 0.001***** | **< 0.001***** | **< 0.001***** | **< 0.001***** | 0.999 |  |  |  |  |  |
| **2017-Nov** | 0.990 | **< 0.001***** | **< 0.001***** | **< 0.001***** | **< 0.001***** | **< 0.001***** | 0.998 | 0.998 |  |  |  |  |
| **2017-Dec** | 0.984 | **< 0.001***** | **< 0.001***** | **< 0.001***** | **< 0.001***** | **< 0.001***** | 0.997 | 0.997 | 0.995 |  |  |  |
| **2018-Jan** | 0.995 | **< 0.001***** | **< 0.001***** | **< 0.001***** | **< 0.001***** | **< 0.001***** | 0.993 | 0.992 | 0.994 | 0.989 |  |  |
| **2018-Feb** | 0.998 | **< 0.001***** | **< 0.001***** | **< 0.001***** | **< 0.001***** | **< 0.001***** | 0.990 | 0.989 | 0.992 | 0.986 | 0.997 |  |

**Notes:**

Table values represent *p*-values from pairwise comparisons of plasma testosterone levels among months in females using one-way analysis of variance (ANOVA) followed by Tukey’s post hoc tests. *** *P* < 0.001, ** *P* < 0.01, * *P* *<* 0.05.

**Table S6 Pairwise comparisons of plasma 17β-estradiol levels among months in females.**

| **Month** | **2017-Mar** | **2017-Apr** | **2017-May** | **2017-Jun** | **2017-Jul** | **2017-Aug** | **2017-Sep** | **2017-Oct** | **2017-Nov** | **2017-Dec** | **2018-Jan** | **2018-Feb** |
| --- | --- | --- | --- | --- | --- | --- | --- | --- | --- | --- | --- | --- |
| **2017-Mar** |  |  |  |  |  |  |  |  |  |  |  |  |
| **2017-Apr** | **< 0.001***** |  |  |  |  |  |  |  |  |  |  |  |
| **2017-May** | **< 0.001***** | 0.079 |  |  |  |  |  |  |  |  |  |  |
| **2017-Jun** | **< 0.001***** | **< 0.001***** | **0.003**** |  |  |  |  |  |  |  |  |  |
| **2017-Jul** | **< 0.001***** | **< 0.001***** | **< 0.001***** | 0.125 |  |  |  |  |  |  |  |  |
| **2017-Aug** | **0.015*** | **< 0.001***** | **< 0.001***** | **< 0.001***** | **< 0.001***** |  |  |  |  |  |  |  |
| **2017-Sep** | 0.508 | **< 0.001***** | **< 0.001***** | **< 0.001***** | **< 0.001***** | **0.002**** |  |  |  |  |  |  |
| **2017-Oct** | 0.465 | **< 0.001***** | **< 0.001***** | **< 0.001***** | **< 0.001***** | **0.002**** | 0.945 |  |  |  |  |  |
| **2017-Nov** | 0.469 | **< 0.001***** | **< 0.001***** | **< 0.001***** | **< 0.001***** | **0.002**** | 0.950 | 0.996 |  |  |  |  |
| **2017-Dec** | 0.550 | **< 0.001***** | **< 0.001***** | **< 0.001***** | **< 0.001***** | **0.003**** | 0.948 | 0.894 | 0.898 |  |  |  |
| **2018-Jan** | 0.719 | **< 0.001***** | **< 0.001***** | **< 0.001***** | **< 0.001***** | **0.006**** | 0.762 | 0.711 | 0.715 | 0.812 |  |  |
| **2018-Feb** | 0.966 | **< 0.001***** | **< 0.001***** | **< 0.001***** | **< 0.001***** | **0.013*** | 0.536 | 0.492 | 0.495 | 0.579 | 0.751 |  |

**Notes:**

Table values represent *p*-values from pairwise comparisons of plasma 17β-estradiol levels among months in females using one-way analysis of variance (ANOVA) followed by Tukey’s post hoc tests. Statistical significance is indicated as follows: *** *P* < 0.001, ** *P* < 0.01, * *P* *<* 0.05.

**Table S7 Pairwise comparisons of plasma progesterone levels among months in females.**

| **Month** | **2017-Mar** | **2017-Apr** | **2017-May** | **2017-Jun** | **2017-Jul** | **2017-Aug** | **2017-Sep** | **2017-Oct** | **2017-Nov** | **2017-Dec** | **2018-Jan** | **2018-Feb** |
| --- | --- | --- | --- | --- | --- | --- | --- | --- | --- | --- | --- | --- |
| **2017-Mar** |  |  |  |  |  |  |  |  |  |  |  |  |
| **2017-Apr** | **< 0.001***** |  |  |  |  |  |  |  |  |  |  |  |
| **2017-May** | **< 0.001***** | **< 0.001***** |  |  |  |  |  |  |  |  |  |  |
| **2017-Jun** | **< 0.001***** | **< 0.001***** | 0.203 |  |  |  |  |  |  |  |  |  |
| **2017-Jul** | **< 0.001***** | 0.261 | **< 0.001***** | **< 0.001***** |  |  |  |  |  |  |  |  |
| **2017-Aug** | **< 0.001***** | **0.010*** | **< 0.001***** | **< 0.001***** | 0.132 |  |  |  |  |  |  |  |
| **2017-Sep** | 0.746 | **< 0.001***** | **< 0.001***** | **< 0.001***** | **< 0.001***** | **< 0.001***** |  |  |  |  |  |  |
| **2017-Oct** | 0.755 | **< 0.001***** | **< 0.001***** | **< 0.001***** | **< 0.001***** | **< 0.001***** | 0.992 |  |  |  |  |  |
| **2017-Nov** | 0.687 | **< 0.001***** | **< 0.001***** | **< 0.001***** | **< 0.001***** | **< 0.001***** | 0.936 | 0.928 |  |  |  |  |
| **2017-Dec** | 0.774 | **< 0.001***** | **< 0.001***** | **< 0.001***** | **< 0.001***** | **< 0.001***** | 0.972 | 0.980 | 0.908 |  |  |  |
| **2018-Jan** | 0.633 | **< 0.001***** | **< 0.001***** | **< 0.001***** | **< 0.001***** | **< 0.001***** | 0.877 | 0.869 | 0.941 | 0.849 |  |  |
| **2018-Feb** | 0.541 | **< 0.001***** | **< 0.001***** | **< 0.001***** | **< 0.001***** | **< 0.001***** | 0.773 | 0.765 | 0.835 | 0.746 | 0.893 |  |

**Notes:**

Table values represent *p*-values from pairwise comparisons of plasma progesterone levels among months in females using one-way analysis of variance (ANOVA) followed by Tukey’s post hoc tests. *** *P* < 0.001, ** *P* < 0.01, * *P* *<* 0.05.
